# Unit Leadership and Climates for Evidence‐Based Practice Implementation in Maternal–Infant Health Units: A Cross‐Sectional Descriptive Study

**DOI:** 10.1111/jan.16531

**Published:** 2024-10-09

**Authors:** Jessica Hsu, Mikayla Morgan, Philip Veliz, Clayton Shuman

**Affiliations:** ^1^ School of Nursing University of Michigan Ann Arbor Michigan USA; ^2^ School of Nursing Duke University Durham North Carolina USA

**Keywords:** evidence‐based practice implementation, maternal–infant health units, maternity nursing, unit climate, unit leadership

## Abstract

**Aims:**

To describe unit leadership and climates for evidence‐based practice implementation and test for differences in unit leader and staff nurses' perceptions within maternal–infant units.

**Design:**

A cross‐sectional descriptive study.

**Methods:**

A convenience sample of maternal–infant unit leaders and nurses (labour, postpartum, neonatal intensive care, paediatrics) from four Midwestern United States hospitals completed a survey including the Implementation Leadership Scale (ILS) and Implementation Climate Scale (ICS). Descriptive statistics described items, subscales and total scores. Independent *t*‐tests with Bonferroni correction tested for differences in perceptions.

**Results:**

A total of 470 nurses and 21 unit leaders responded, representing 17 units. Ratings of unit leadership and climates for implementation were modest at best [ICS: *M* = 2.17 (nurses), 2.41 (leaders); ILS: *M* = 2.4 (nurses), 2.98 (leaders)]. Unit leader ratings were statistically significant and higher than nurse ratings.

**Conclusion:**

This study is one of the first to describe unit leadership and climates for implementation in maternal–infant health. To improve outcomes and equity in maternal–infant health, attention on leadership behaviours and unit climates for evidence‐based practice implementation is needed.

**Implications for the Profession:**

Nurse leaders are encouraged to evaluate their leadership behaviours and the unit climates they facilitate, and work to improve areas of concern or where staff perceptions differ. Staff nurses should work with their leaders to identify resources and rewards/recognition which support and facilitate EBP implementation.

**Impact:**

This study addressed a gap in research examining the social dynamic factors of unit leadership and climate for evidence‐based practice implementation in maternal–infant units. Leadership behaviours for implementation and unit climate were rated moderately by both staff and leaders. Unit leaders rated their implementation leadership and climates higher in almost all items. This study is relevant to unit leaders and nurses in maternal–infant units in the United States.

**Reporting Method:**

This study adhered to STROBE guidelines.

**Patient or Public Contribution:**

No patient or public contribution.

**Summary**

- What does this paper contribute to the wider global clinical community?
  ○ This paper adds to the literature by exploring the social dynamic factors of evidence‐based practice implementation in maternal–infant units.

## 1 Introduction

Maternal morbidity and mortality is a public health crisis in the United States, where outcomes are the worst among developed countries (Douthard et al. 2021). Implementing evidence‐based practices (EBPs) is known to increase the quality of care, patient safety and reduce inequities (Connor et al. 2023). However, implementation can be challenging, and it is estimated to take up to 17 years for research evidence to reach clinical practice and benefit patients (Morris, Wooding, and Grant 2011). Numerous context factors affect the implementation and sustained use of EBPs such as structural and social dynamic factors (Shuman et al. 2023). Structural factors refer to the physical or operational aspects of a setting that either facilitate or hinder the implementation of EBP, while social dynamic factors encompass the roles, relationships and interactions among individuals and groups within a practice setting, including unit leadership and climate (Shuman et al. 2023). Although the organisational and implementation science literatures acknowledge the importance of leadership and climate to implementation, few studies in maternal–infant health have described these important factors.

## 2 Background

Leadership is an important component of the organisational process as it influences many contextual factors, which are important for creating an environment conducive to change and strengthening the performance of healthcare systems and units (Li et al. 2018). For example, transformational leadership in particular has been found to support innovation and promote implementation and use of EBPs (Aarons et al. 2015). Leadership behaviours for EBP implementation is defined as a specific and strategic approach to leadership characterised by a set of influencing behaviours leading to positive outcomes for the implementation of EBPs (Aarons, Ehrhart, and Farahnak 2014; Shuman et al. 2020). These behaviours play a critical role in creating a unit climate that is conducive to EBP implementation, ultimately resulting in patients receiving care that is informed by the best available evidence (Shuman et al. 2018, 2019).

Unit climate for EBP implementation is defined as the meaning nurse leaders and nurses attach to the policies, practices and procedures they experience and the behaviours they see being rewarded, supported and expected related to EBP implementation (Ehrhart, Aarons, and Farahnak 2014). Unit climate for EBP implementation is crucial as it reflects the value and priority placed on EBP within the unit (Ehrhart, Aarons, and Farahnak 2014). A positive unit climate is an indicator of strong support for EBP among staff and leadership, which can significantly increase the likelihood of successful implementation. On the other hand, a negative or indifferent climate may hinder EBP implementation efforts, as it may suggest a lack of commitment or interest in implementing and using EBPs (Aarons et al. 2014). Therefore, fostering a positive unit climate that emphasises the importance of EBP is essential for promoting its successful implementation and integration into clinical practice (Cassidy, Flynn, and Shuman 2021).

Despite the importance of implementation climate and leadership to implementation, few studies have examined the social dynamic factors of unit leadership and climate for EBP implementation. A previous study addressed this gap by examining unit leadership and climates for EBP implementation, but it was done specifically in the context of adult medical–surgical nursing units (Shuman et al. 2019). Implementation leadership behaviours of nursing unit managers are associated with unit climates for EBP implementation (Shuman et al. 2018). However, misalignment of staff and leader ratings of implementation leadership behaviours are associated with worse unit climates for EBP implementation (Shuman et al. 2023). Considering the crisis state of maternal morbidity and mortality in the United States and the lack of attention to understanding these contextual factors within maternal–infant health, it is important to examine these factors in maternal–infant health units.

## 3 The Study

### 3.1 Aims

This study aims to add to the literature by examining unit leadership and climates for implementation in maternal–infant nursing units. The objectives of this study were to (1) describe unit leadership and climates for EBP implementation and (2) test for differences in unit leader and staff nurses' perceptions of EBP implementation leadership behaviours and unit climates for EBP implementation within maternal–infant units.

The following research questions guided the study:

1. How do maternal–infant nurses and unit leaders perceive unit leadership and climates for EBP implementation?
2. Are there any significant differences in unit leader and staff nurses' perceptions of EBP implementation leadership behaviours and unit climates for EBP implementation within maternal–infant units?

## 4 Methods

### 4.1 Design

A multi‐site cross‐sectional descriptive design was used to address the aims. The ethics review board at the University of Michigan and each participating hospital approved this study prior to data collection in 2020.

### 4.2 Setting

A convenience sample of four Midwestern United States hospitals located in counties with high prevalence of opioid use disorder and overdose deaths were recruited. Hospitals were located in Michigan, Ohio, Illinois and Minnesota. From these hospitals, 17 units were identified that provided care to birthing individuals, postpartum individuals and/or infants (labour, postpartum, paediatric, neonatal intensive care).

### 4.3 Sample

#### 4.3.1 Unit Leaders

Unit leaders were defined as registered nurses who oversaw unit‐level operations and were responsible for care delivered by clinical staff. Inclusion criteria were: (a) licensed as a registered nurse; (b) had responsibility for unit‐level operations; (c) was a direct supervisor of nursing staff on a study unit; and (d) was not serving as an interim. At least one unit leader from each of the 17 units was invited. Four units had a dual or team leadership model, in which case all leaders meeting eligibility criteria were invited.

#### 4.3.2 Staff Nurses

Staff nurses were defined as licensed registered nurses providing direct patient care on a study unit. Inclusion criteria were: (a) licensed as a registered nurse; (b) worked ≥ 0.40 full‐time equivalents; (c) provides direct patient care; and (d) designated as staff on a study unit. Those who were designated as contingency, agency staff or floated to other units were excluded.

### 4.4 Study Variables and Measures

#### 4.4.1 Demographic Data

Demographic data collected from participants included age, sex, race, education level and shift type (e.g., days, nights), years of experience as a registered nurse, years of experience as a unit leader and years of experience as a registered nurse or unit leader in the current hospital and unit.

#### 4.4.2 Unit Leadership Behaviours for EBP Implementation

The 12‐item Implementation Leadership Scale (ILS) was used to measure unit leaders' self‐perceptions and staff nurses' perceptions of their unit leaders' leadership behaviours for EBP implementation in four domains: (a) proactive leadership (three items), (b) knowledgeable leadership (three items), (c) supportive leadership (three items), and (d) perseverant leadership (three items) (Aarons, Ehrhart, and Farahnak 2014). Respondents indicated their level of agreement with each item using a Likert scale from 0 to 4 (0 = not at all; 1 = slight extent; 2 = moderate extent; 3 = great extent; 4 = very great extent). The ILS total score was calculated by summing the scores for each of the 12 items and dividing by 12 to obtain the mean. Subscale scores were determined by adding the response value for each item within a subscale and dividing by the number of subscale items to obtain a mean. The ILS has demonstrated reliability and validity among samples of nurses and nursing unit leaders (Hu et al. 2021; Llarena et al. 2023; Mandrou, Tsounis, and Sarafis 2020; Shuman et al. 2020). In an acute care nursing context, the ILS previously demonstrated excellent reliability (Cronbach's *α* = 0.91–0.98), and construct validity has previously been demonstrated through confirmatory factor analysis using two independent samples (Shuman et al. 2020).

#### 4.4.3 Unit Climate for EBP Implementation

The 18‐item Implementation Climate Scale (ICS) was used to measure the extent to which employees perceive their unit to support EBP implementation in six domains: (a) focus on EBP (three items), (b) educational support for EBP (three items), (c) recognition for EBP (three items), (d) rewards for EBP (three items), (e) selection (hiring staff) for EBP (three items) and (f) selection (hiring staff) for openness (three items) (Ehrhart, Aarons, and Farahnak 2014). Respondents indicated their level of agreement with each item using a Likert scale from 0 to 4 (0 = not at all; 4 = very great extent). The ICS total score was calculated by summing all 18 items and dividing by 18. Subscale scores were determined by summing the response values for each item within a subscale and dividing by the number of subscale items. The ICS has demonstrated validity and reliability among nurse and nursing unit leader samples in previous studies (Ehrhart et al. 2021; Shuman et al. 2018, 2019). Ehrhart et al. (2021) demonstrated the ICS's reliability in two samples of nurses, (Cronbach's *α* = 0.94–0.95) as well as construct validity using confirmatory factor analysis among two independent samples.

### 4.5 Study Procedure and Data Collection

Site coordinators assisted with data collection and were trained using a detailed manual and face‐to‐face training session. Site coordinators helped to identify eligible nurses and unit leaders and assisted with questionnaire distribution. Electronic surveys via Qualtrics were used to collect the data from participants. The identified eligible nurses and unit leaders were sent an email inviting them to complete a web‐based survey inclusive of the ILS, ICS and demographic items. Nurses and unit leaders were provided with a study information document explaining the study, their involvement, potential risks and benefits, and providing contact information for the study team. Participants were informed that completion and submission of the survey signified their consent to participate. Email reminders and a $10 cash card incentive were used to thank participants for their time and effort. Survey data collection occurred over the course of 1 month.

### 4.6 Statistical Analysis

Data were analysed in Stata version 18.0 (StataCorp. 2023). Missing values were explored to identify patterns. If respondents completed less than 50% of a scale (ILS or ICS), their responses for that scale were listwise deleted. If less than 50% of a subscale was completed, their responses for that subscale were listwise deleted from subscale analyses. Scale reliability among unit leaders' and staff nurses' was evaluated using Cronbach's *α*. Descriptive statistics (mean and standard deviation) described items, subscales, and total scores for the ILS and the ICS for unit leaders and nurses. Independent *t*‐tests (*α* = 0.05) were used to test for differences in perceptions between staff nurse and unit leader scores. Bonferroni correction was used to reduce Type 1 errors, leading to a new significance level of *p* < 0.01 for the ILS and 0.007 for the ICS. Comparisons at or below these corrected values were considered statistically significant.

### 4.7 Ethical Considerations

This study was deemed exempt by the University of Michigan Health Sciences and Behavioural Sciences Institutional Review Board on October 18, 2019 (ethical approval number: HUM00171191).

## 5 Results

### 5.1 Participants

The sample included 21 unit leaders (87.5% response rate) and 470 staff nurses (50.5% response rate). Seventeen units were represented by the respondents (labour and delivery, *N* = 4; neonatal intensive care, *N* = 4; postpartum, *N* = 4; paediatrics, *N* = 5). Demographic characteristics of unit leaders and staff nurses are described in Table 1. The majority of unit leaders and staff nurses were White (61.9% and 71.6%, respectively) and female (95.2% and 88.7%, respectively). Most unit leaders had a Master's (66.7%) or Bachelor's (33.3%) degree. Most staff nurses were Bachelor's prepared (58.9%). The average years of experience as a registered nurse was 16.5 years for staff nurses and 19.6 years for unit leaders. Additional demographic characteristics are described in Table 1.

**TABLE 1:** Respondents' demographic and professional information (*N* = 491).

	Unit leader (*N* = 21)		Staff nurse (*N* = 470)	
	*M*	SD	*M*	SD
Age (years)	38.61	7.52	37.87	10.99
Years as RN	19.57	8.37	16.54	9.56
Years as unit leader	9.14	3.95		
Years in role in current hospital	8.81	3.79	13.66	9.35
Years in role in current unit	8.00	3.16	12.28	8.75
	*n*	%	*n*	%
Sex				
Female	20	95.24	417	88.72
Male	1	4.76	8	1.70
Prefer not to say			6	1.28
Missing			39	8.30
Race				
African American/Black	2	9.52	23	4.89
Asian	3	14.29	12	2.55
Hispanic	1	4.76	18	3.83
Middle Eastern/North African			1	0.21
Native American/Alaska Native			1	0.21
Native Hawaiian/Other Pacific Islander				
White	13	61.90	332	70.64
Multiple races	2	9.52	15	3.19
Prefer not to say/Missing			68	14.47
Education				
Diploma			10	2.13
Associate's			93	19.79
Bachelor's	7	33.33	277	58.94
Master's	14	66.67	46	9.79
Doctorate			3	0.64
Missing			41	8.72
Shift				
Days			216	45.96
Evenings			6	1.28
Nights			176	37.45
Rotate			33	7.02
Missing			39	8.30

### 5.2 Unit Leader EBP Leadership Behaviours

The ILS was completed by 446 staff nurses and 21 unit leaders. The scale reliability was good to excellent for staff nurses (total, *α* = 0.97; subscales, *α* = 0.90–0.94) and unit leaders (total, *α* = 0.93; subscales, *α* = 0.81–0.90). The mean total ILS score was 2.41 (SD = 1.01) for staff nurses and 2.98 (SD = 0.88) for unit leaders. Of the four subscales, proactive leadership had the lowest mean score among both staff nurses (*M* = 2.25; SD = 1.01) and unit leaders (*M* = 2.49; SD = 1.01). For proactive leadership, about 33% of staff nurses rated the item ‘removed obstacles to implementation of EBP’ highly (score of 3 or 4) while 52% of unit leaders rated it highly. Supportive leadership was rated the highest subscale at 2.54 (SD = 1.00) for staff nurses and 3.62 (SD = 0.58) for unit leaders. Approximately 95% of unit leaders rated themselves highly (3–4) on all three items associated with this subscale. Approximately 50%–57% of staff nurses rated supportive leadership items highly as well. Subscale scores of knowledgeable, supportive and perseverant leadership were significantly different (Bonferroni corrected *p* values < 0.01) between staff nurses and unit leaders, with unit leaders scoring themselves higher on average than staff nurses for each subscale. Total scores were also significantly different between staff nurses and unit leaders (*t* = −8.74; *p* < 0.01), with unit leaders rating their own leadership behaviours for EBP implementation higher than staff ratings (Table 2).

**TABLE 2:** Staff nurse and unit leader ratings of implementation leadership.

	Staff nurse							Unit leader								
	*N*	α	*M*	SD	Mdn	IQR	Rating 3–4 (%)	*N*	α	*M*	SD	Mdn	IQR	Rating 3–4 (%)	*t*	*p* *
Subscale 1: proactive		0.90	2.25	1.01	2.33	1.67–3.00			0.84	2.49	1.01	2.66	2.00–3.00		−1.84	0.07
Developed a plan to facilitate EBP implementation	445		2.30	1.01	2.00	2.00–3.00	45.84	21		2.48	0.87	3.00	2.00–3.00	52.38		
Removed obstacles to implementation of EBP	442		2.10	0.97	2.00	1.00–3.00	33.48	21		2.43	0.98	3.00	2.00–3.00	52.38		
Established clear department standards for implementation	444		2.36	1.03	2.00	2.00–3.00	46.17	21		2.57	1.21	3.00	2.00–3.00	57.14		
Subscale 2: knowledgeable		0.94	2.47	1.01	2.66	2.00–3.00			0.81	2.90	0.67	2.66	2.67–3.00		−3.32	< 0.001
Is knowledgeable about EBP	445		2.57	0.98	3.00	2.00–3.00	56.40	21		3.00	0.63	3.00	3.00–3.00	80.95		
Is able to answer staff questions about EBP	446		2.35	1.03	2.00	2.00–3.00	46.86	21		2.81	0.75	3.00	2.00–3.00	61.90		
Knows what he or she is talking about when it comes to EBP	445		2.50	1.00	3.00	2.00–3.00	53.26	20		2.9	0.64	3.00	2.00–3.00	75.00		
Subscale 3: supportive		0.91	2.54	1.00	2.66	2.00–3.00			0.90	3.62	0.58	3.66	3.67–4.00		−8.50	< 0.001
Recognises and appreciates employee efforts	443		2.44	1.04	3.00	2.00–3.00	50.11	21		3.48	0.60	4.00	3.00–4.00	95.24		
Supports employee efforts to learn more about EBP	445		2.58	0.99	3.00	2.00–3.00	55.51	21		3.67	0.58	4.00	3.00–4.00	95.24		
Supports employee efforts to use EBP	443		2.59	0.97	3.00	2.00–3.00	57.79	21		3.71	0.56	4.00	4.00–4.00	95.24		
Subscale 4: perseverant		0.93	2.38	0.99	2.33	1.67–3			0.82	2.89	0.79	3.00	2.33–3.33		−4.05	< 0.001
Perseveres through the ups and downs of implementing EBP	443		2.38	0.99	2.00	2.00–3.00	48.08	21		2.90	0.83	3.00	2.00–3.00	71.43		
Carries on through the challenges of implementing EBP	446		2.40	0.99	3.00	2.00–3.00	50.67	21		2.90	0.70	3.00	2.00–3.00	71.43		
Reacts to critical issues regarding implementation of EBP	444		2.35	1.00	2.00	2.00–3.00	45.27	21		2.86	0.85	3.00	3.00–3.00	76.19		
Total score		0.97	2.41	1.01	2.50	1.92–3.00			0.93	2.98	0.88	3.00	2.67–3.25		−8.74	< 0.001

*Note:* Scale range is 0–4 (0 = not at all; 1 = slight extent; 2 = moderate extent; 3 = great extent; 4 = very great extent).

Abbreviations: EBP, evidence‐based practice; IQR, interquartile range; Mdn, median.

* Bonferroni corrected, *p* < 0.01.

### 5.3 Unit Climate for EBP Implementation

The ICS was completed by 470 staff nurses and 21 unit leaders. The scale reliability was acceptable to excellent for staff nurses (total, *α* = 0.93; subscales, *α* = 0.72–0.84) and acceptable to good for unit leaders (total, *α* = 0.86; subscales, *α* = 0.64–0.88), with the exception of the rewards for EBP subscale which had low reliability (*α* = 0.23). The total mean ICS score for staff nurses was 2.17 (SD = 1.19) and 2.41 (SD = 1.24) for unit leaders. The highest rated subscale was Focus on EBP for both staff nurses (*M* = 2.76, SD = 0.92) and unit leaders (*M* = 3.02, SD = 0.98). Rewards for EBP were the lowest for both staff nurses (Mdn = 0.66, IQR = 0.00–1.33) and unit leaders (Mdn = 1.33, IQR = 0.67–1.67). The median and interquartile range were used to describe the rewards for EBP subscale because the standard deviation of the subscale was larger than the mean. Subscale scores for selection for openness were significantly different between staff nurses and unit leaders (*p* < 0.001). Educational support for EBP was the only subscale in which staff nurses scored unit leaders higher for all individual items. About 40% of staff nurses rated the item ‘unit provides conferences, workshops or seminars’ highly, while only about 23% of unit leaders agreed with that statement. The total ICS score was found to significantly differ between staff nurses and unit leaders (*t* = −3.78, *p* < 0.001), with unit leaders rating the unit climate higher than staff nurses (Table 3).

**TABLE 3:** Staff nurse and Unit leader ratings of Implementation Climate.

	Staff nurse							Unit leader								
	*N*	α	*M*	SD	Mdn	IQR	Rating 3–4 (%)	*N*	α	*M*	SD	Mdn	IQR	Rating 3–4 (%)	*t*	*p* *
Subscale 1: focus on EBP		0.83	2.76	0.92	3.00	2.33–3.33			0.64	3.02	0.98	3.00	2.67–3.67		−2.18	0.04
One of my unit's goals is to use EBP effectively	467		2.76	0.92	3.00	2.00–3.00	66.17	21		3.10	1.04	3.00	3.00–4.00	85.71		
People think implementation is important	469		2.81	0.91	3.00	2.00–3.00	65.25	21		2.86	0.91	3.00	2.00–4.00	61.90		
Using EBP is a top priority in this unit	466		2.71	0.93	3.00	2.00–3.00	61.16	21		3.10	1.00	3.00	3.00–4.00	76.19		
Subscale 2: educational support for EBP		0.81	2.27	1.08	2.33	1.67–3.00			0.69	2.21	1.08	2.00	1.67–3.00		0.47	0.64
My unit provides conferences, workshops, or seminars	465		2.17	1.14	2.00	1.00–3.00	40.86	21		1.90	1.09	2.00	1.00–2.00	23.81		
My unit provides EBP trainings or in‐services	467		2.46	1.00	2.00	2.00–3.00	49.46	21		2.33	1.20	2.00	2.00–3.00	42.86		
My unit provides EBP training materials, journals, etc.	465		2.18	1.08	2.00	1.00–3.00	39.78	21		2.38	0.92	2.00	2.00–3.00	47.62		
Subscale 3: recognition for EBP		0.72	2.10	1.17	2.00	1.67–2.67			0.72	2.41	1.17	2.33	1.67–3.00		−2.05	0.28
Clinicians who use EBP are seen as clinical experts	467		2.41	0.99	3.00	2.00–3.00	50.96	21		2.71	1.10	3.00	2.00–4.00	61.90		
Clinicians who use EBP are held in high esteem	466		2.39	1.02	2.00	2.00–3.00	49.36	21		2.71	0.85	3.00	2.00–3.00	57.14		
Clinicians who use EBP are more likely to be promoted	465		1.48	1.23	2.00	0.00–2.00	23.87	21		1.81	1.33	2.00	0.00–3.00	38.10		
Subscale 4: rewards for EBP		0.74	0.95	1.16	0.66	0.00–1.33			0.23	1.19	1.41	1.33	0.67–1.67		−1.59	0.19
My unit provides financial incentives for use of EBP	462		0.67	1.02	0.00	0.00–1.00	8.01	21		0.62	1.02	0.00	0.00–1.00	9.52		
Staff who use EBP are more likely to get a bonus/raise	464		0.76	1.07	0.00	0.00–1.00	8.41	21		0.67	1.15	0.00	0.00–1.00	9.52		
My unit provides the ability to accumulate compensated time for the use of EBPs	463		1.42	1.24	1.00	0.00–2.00	22.68	21		2.29	1.38	3.00	1.00–3.00	61.90		
Subscale 5: selection for EBP		0.81	2.38	0.98	2.33	2.00–3.00			0.72	2.63	0.81	2.33	2.33–3.00		−2.01	0.02
My unit selects staff who have previously used EBP	467		2.23	0.96	2.00	2.00–3.00	39.19	21		2.48	0.81	2.00	2.00–3.00	47.62		
My unit selects staff who have formal education supporting EBP	467		2.35	1.02	2.00	2.00–3.00	47.75	21		2.52	0.81	3.00	2.00–3.00	52.38		
My unit selects staff who value EBP	469		2.57	0.93	3.00	2.00–3.00	54.16	21		2.90	0.77	3.00	2.00–3.00	66.67		
Subscale 6: Selection for Openness		0.84	2.55	0.85	2.66	2.00–3.00			0.88	2.98	0.96	3.00	2.67–3.67		−3.96	< 0.001
My unit selects staff who are adaptable	470		2.61	0.83	3.00	2.00–3.00	58.51	21		3.10	0.89	3.00	3.00–4.00	76.19		
My unit selects staff who are flexible	469		2.51	0.85	3.00	2.00–3.00	51.60	21		3.00	1.10	3.00	2.00–4.00	66.67		
My unit selects staff who are open to new types of interventions	467		2.52	0.88	3.00	2.00–3.00	52.46	21		2.86	0.91	3.00	2.00–3.00	71.43		
Total score		0.93	2.17	1.19	2.16	1.92–3.00			0.86	2.41	1.24	2.38	2.00–2.67		−3.78	< 0.001

*Note:* Scale range is 0–4 (0 = not at all; 1 = slight extent; 2 = moderate extent; 3 = great extent; 4 = very great extent).

Abbreviations: EBP, evidence‐based practice; IQR, interquartile range; Mdn, median.

* Bonferroni corrected, *p* < 0.07.

## 6 Discussion

This study is one of the first to describe leadership behaviours and climates for EBP implementation within maternal–infant health nursing units. Describing and examining these important contextual factors known to affect implementation may help to identify context‐specific barriers and facilitators to EBP implementation within maternal–infant health and inform strategies to address them. By improving the context for implementation, more EBPs may be effectively implemented resulting in improvements to healthcare delivery, patient outcomes and equity in maternal–infant health.

Unit leadership behaviours were generally rated as moderate by both staff nurses and unit leaders. Similar findings were noted by Shuman et al. (2019) among staff nurses in China. Efforts to improve leadership behaviours for EBP implementation are warranted. Notably, supportive leadership had the greatest difference in perception, with unit leaders rating themselves more than one full point above staff ratings. This difference in perception may be due to numerous factors. For example, unit leaders are more likely to be aware of the policies, practices and procedures in the unit as well as the extent to which these support implementation in the larger organisation (Shuman et al. 2023). It could also be that unit leaders were not providing the support their nurses desired or required in regards to implementing and using EBPs. This difference between staff nurses and unit leaders regarding Supportive Leadership in maternal–infant health units was not found in a previous study of staff nurses and unit leaders in adult medical–surgical units (Shuman et al. 2019). Additionally, the knowledgeable and perseverant subscales were also significantly different, as was the total ILS score, with unit leaders rating themselves higher on all. This may affect the implementation of EBP in maternal–infant units as unit leaders may overestimate their leadership behaviours which could adversely affect their effectiveness as a leader (Aarons et al. 2017). Further research should examine the potential effect and severity these perceptual differences have on implementation and patient outcomes.

Similar to leadership behaviours, implementation climate was rated moderately across most subscales and overall. Previous studies in adult medical–surgical units (Shuman et al. 2019) and critical care units (Boehm et al. 2022) reported similar scores across subscales and overall, including the lowest ratings for the Rewards for EBP subscale. Unit leader and staff nurse ratings of climate overall differed significantly, as did the Selection for Openness subscale. For five of the six subscales, ratings were relatively similar for both samples. Among medical–surgical nurses, Shuman et al. (2019) did not observe significant differences between staff and leaders on the ICS nor any of its subscales. In the current study, a difference in perception was observed for the item, “my unit provides conferences, workshops, or seminars,” in which over 40% of staff nurses rated the item positively (“great extent” or “very great extent”) compared to 23% of unit leaders. The reason for this difference in perception is unknown; however, staff nurses may have assumed that in‐services or unit meetings were included in this item or are aware of other educational support and resources not known to leadership or provided by leadership. Regardless, both groups rated this item relatively low suggesting that future work should focus on this domain. Selection for openness was the only ICS subscale that significantly differed among staff and leaders. This subscale measures the extent to which a unit hires new staff who are open to using new innovations and practices. An important contributor to successful implementation is staff willingness to change clinical practice to align with EBP. A potential explanation for this difference in perception may be related to interviewing and hiring processes in which unit leaders may take a more prominent role. It is likely challenging for unit leaders to accurately and reliably assess a candidate's openness to EBP within the context of an interview.

Findings from this study are immediately relevant to research and practice. More research is needed to better understand the effect of these contextual factors on implementation and patient outcomes. Further, additional efforts are needed to develop and test pragmatic interventions to improve these contextual factors. For example, the Leadership for Organisational Change and Implementation (LOCI) program, designed to improve leadership and climate for implementation, has been tested in mental health clinics (Aarons et al. 2015; Skar et al. 2022) and may be adaptable and effective within nursing contexts. Unit leaders are encouraged to assess their leadership behaviours and unit climate for EBP implementation to identify specific areas for improvement. Attention to the implementation context is often applied during pre‐implementation stages as part of a context assessment to identify facilitators and barriers. However, as Cassidy, Flynn, and Shuman (2021) argue, devoting attention to these factors outside of a planned implementation project may help to facilitate the development of conducive implementation contexts thus providing a more desirable and prepared context for numerous implementation projects.

### 6.1 Limitations

This study has numerous limitations. A convenience sample of four Midwestern United States hospitals was used, which may limit generalizability. In an effort to support generalizability, the four hospitals included in this study varied in size, geographic location, type (e.g., academic medical centre, community hospital) and the racial and ethnic communities served. Overall, the ILS and ICS demonstrated good to excellent reliability in our study. Three of the six ICS subscale had relatively lower reliability among unit leaders (*α* < 0.70) compared to staff nurses, which could be due to the small sample of unit leaders in this study (*N* = 21). Specifically, the rewards for EBP subscale had the lowest reliability for unit leaders (*α* = 0.23) and thus these results should be interpreted carefully. It is important to note that data were collected during the COVID‐19 pandemic which may or may not have affected perceptions of unit leadership and climates. Although there is a chance for bias when using an incentive, the incentive amount was minimal ($10) and was offered only as a token of appreciation for the participants' time and effort.

## 7 Conclusion

Improving maternal–infant health in the United States requires efficient and effective implementation of EBPs. Implementation of EBPs is greatly affected by contextual factors. This study found that leadership behaviours and unit climates for EBP implementation in maternal–infant health units were moderate at best, suggesting the need for improvement and increased attention. Future studies are warranted to examine leadership and climate in other geographic regions and identify associations with implementation and patient outcomes. Nurse leaders are encouraged to evaluate their leadership behaviours and the unit climates they facilitate, and work to improve areas of concern or areas where they differ with staff perceptions. Staff nurses are encouraged to work with their unit leaders to identify effective resources and rewards/recognition which support and facilitate EBP implementation in maternal–infant health.

## Author Contributions

All the authors have made substantial contributions to conception and design, or acquisition of data, or analysis and interpretation of data; involved in drafting the manuscript or revising it critically for important intellectual content; given final approval of the version to be published. Each author should have participated sufficiently in the work to take public responsibility for appropriate portions of the content; agreed to be accountable for all aspects of the work in ensuring that questions related to the accuracy or integrity of any part of the work are appropriately investigated and resolved.

## Conflicts of Interest

The authors declare no conflicts of interest.

## Peer Review

The peer review history for this article is available at https://www.webofscience.com/api/gateway/wos/peer‐review/10.1111/jan.16531.

## Data Availability

The data that support the findings of this study are available from the corresponding author upon reasonable request.
